# The impact of basic pension for urban and rural residents on the subjective well-being of the older adult in Chinese rural areas

**DOI:** 10.3389/fpubh.2024.1394688

**Published:** 2024-05-20

**Authors:** Jianhai Yang, Ziying Li, Jiexin Zhang, Zheng Zang

**Affiliations:** ^1^School of Economics, Beijing Technology and Business University, Beijing, China; ^2^School of Marxism, Soochow University, Suzhou, China

**Keywords:** basic pension insurance for urban and rural residents, basic pension, older adult in rural areas, subjective well-being, degree of depression

## Abstract

**Introduction:**

As an important component of the social security system, basic pension insurance for urban and rural residents is expected to improve the quality of life of rural older adult people and make their lives better and happier. This article mainly studies the relationship between the basic pension for urban and rural residents and the subjective well-being of older adult people in rural China.

**Methods:**

This paper uses data from the China Health and Retirement Longitudinal Study (CHARLS) for the years 2018 and 2020. It selected samples of rural older adult people aged 60 and above, ultimately obtaining 9,310 samples. The impact of the basic pension for urban and rural residents on the subjective well-being of rural older adult people was estimated by constructing Ordinary Least Squares (OLS) estimation methods and ordered logistic regression models. The robustness of the results was verified by changing the regression methods, and the samples were divided into different groups for heterogeneity analysis according to three different standards.

**Results:**

The results show that the basic pension for urban and rural residents significantly improves the life satisfaction of rural older adult, reduces their degrees of depression, and thereby enhances their subjective well-being. The impact of the basic pension for urban and rural residents is more significant for older adult individuals in areas with a higher gender ratio, those suffering from chronic diseases, and those in the eastern regions of the country. Further verification indicates that the basic pension for urban and rural residents enhances the subjective well-being of the rural older adult by improving their health status and reducing their labor supply.

**Discussion:**

Most of the existing research on basic pension insurance for urban and rural residents and subjective well-being has been conducted from the perspective of whether individuals are enrolled in the pension scheme or whether they received a pension. However, there are few studies analyzing from the perspective of the amount of pension benefits received by residents. The results of this study help to enrich the research perspective on the basic pension insurance system for urban and rural residents in China and expand the understanding of the impact and value of the basic pension for urban and rural residents.

## 1 Introduction and literature review

With the increasing degree of population aging, how to deal with the older adult care issue has become a focus of attention in China. In rural China, the older adult care for most older adult people is still primarily based on family care (1), but the development and changes in society, along with changes in family structure, have continuously increased the burden of family care (2), limiting the function of family care. Therefore, to better guarantee the older adult care life of rural residents, China implemented the New Rural Pension Insurance System in 2009 and merged it with the Urban Resident Pension Insurance into the Urban and Rural Residents Pension Insurance System in 2014, to serve as a supplement to the family care of urban and rural residents.

After the implementation of the basic pension insurance system for urban and rural residents, many domestic scholars have evaluated the impact of this policy on the living conditions and welfare levels of the older adult in rural areas and their families. Some studies indicate that basic pension insurance for urban and rural residents indeed improves the income level of the older adult in rural areas (3), promotes consumption (4), and reduces older adult poverty (5). However, most scholars focus only on the economic welfare aspect, and only a few scholars pay attention to its subjective welfare effects. Existing research points out that participation in basic pension insurance for urban and rural residents can significantly enhance the subjective well-being of the older adult in rural areas. Specifically, on the one hand, the basic pension insurance for urban and rural residents provides an economic source for the older adult, reduces expected risks, and decreases their uncertainty about the future, thereby enhancing well-being (6); on the other hand, by playing a role in income redistribution (7), the basic pension insurance for urban and rural residents alleviates the negative impact of income disparities on well-being, thus enhancing happiness (8). Therefore, studying the relationship between the basic pension for urban and rural residents and the subjective well-being of the older adult in rural areas is of great significance for improving the quality of older adult care for rural residents.

Subjective well-being is the emotional and cognitive overall evaluation of one’s life quality based on subjective experiences of current life (9). By assessing subjective well-being, it is possible to understand the effects of public policy implementation and whether there is an increase in public satisfaction (10). Variables of subjective well-being include broad measures of overall life satisfaction, as well as satisfaction in narrower areas such as household income (11). A review of previous studies finds that explorations into the factors affecting older adult subjective well-being mainly focus on individual characteristics and social characteristics (12). Looking at the factors of individual characteristics, the impact of gender on subjective well-being has not reached a consistent conclusion (13, 14), a study in South Africa found that women rise first and then fall, while men do the opposite (15). There is a U-shaped relationship between age and subjective well-being, and subjective well-being tends to decrease with age before increasing (13, 16). Variables such as health status, education level, and the number of children have a positive correlation with subjective well-being (17–20). In terms of social characteristics, securing an income level can improve the welfare of the older adult (21), and the relationship between income level and subjective well-being is an inverted U-shape, where subjective well-being increases with income to a maximum point and then declines (22, 23). Variables such as unemployment and environmental issues have a negative impact on well-being (24–26), while factors like social relationships are positively correlated with well-being (27).

Moreover, some studies both domestically and internationally have shown that there exists a significant positive relationship between pension systems and the subjective well-being of the older adult (28–31), with pensions providing a reliable source of income for the older adult, reducing the insecurity felt about pensions due to individual resource and economic condition differences, thereby enhancing subjective well-being (32). The level of pension income affects the subjective well-being of the older adult (33), and a generous pension can significantly improve the subjective well-being of the older adult and reduce the incidence of diagnosed depression (34). For the older adult in rural China, pensions have a significant impact on their level of well-being (35), with basic pension insurance for urban and rural residents being one of their main sources of pension income. By the end of 2022, the number of participants in the basic pension insurance for urban and rural residents had reached 549.52 million, essentially achieving full coverage for rural residents. From this, it can be seen that the subjective welfare effects produced by the basic pension insurance for urban and rural residents have a certain impact on the subjective well-being of the older adult.

Although the basic pension insurance system for urban and rural residents has been implemented for over a decade, its level of protection remains very limited, with the actual pension replacement rate far below the target level (36), unable to provide the older adult with sufficient pension security. Looking at the actual operation of the system, due to the weak payment ability of most older adult people in rural areas or their complete lack of ability to pay, the pensions they receive is mainly based on basic pension. Furthermore, the basic pension for urban and rural residents also faces issues such as low benefit levels and slow increase rates, which have not yet achieved the goal of “ensuring basic needs” (37, 38). In this context, it is necessary to increase the level of basic pensions for urban and rural residents to ensure the basic living of the older adult in rural areas, alleviate the burden of family care, and enhance the subjective well-being of the older adult.

In summary, current research has proven that participation in basic pension insurance for urban and rural residents can significantly enhance the subjective well-being of the older adult (39, 40). However, existing studies on the basic pension insurance for urban and rural residents and the subjective well-being of rural older adult people have mostly focused on whether they participate or receive pensions, without much discussion on the relationship between the level of basic pensions and happiness. Therefore, this paper utilizes microdata from the China Health and Retirement Longitudinal Study (CHARLS) for the years 2018 and 2020, constructs OLS and ordered logistic regression models, explores the impact of the basic pension for urban and rural residents on the subjective well-being of rural older adult people, and conducts a mechanism analysis, which is of certain practical significance.

This paper focuses on answering the following three questions: What is the impact of the basic pension for urban and rural residents on the subjective well-being of the rural older adult? How do these effects differ among the older adult of different genders, income levels, and regions? How does the basic pension for urban and rural residents affect the well-being of the older adult? The paper will make contributions in the following two areas: (1) By combining the amount of the basic pension for urban and rural residents with micro data to explore the impact of the basic pension on subjective well-being, and further analyzing the heterogeneity of the impact; (2) Using the mediating effect method to test the impact mechanism of the subjective welfare effect of the basic pension for urban and rural residents.

## 2 Materials and methods

### 2.1 Conceptual framework

The primary focus of this paper is the impact of basic pension for urban and rural residents on the subjective well-being of the older adult in rural areas. The outcome variables selected for this study are the degree of depression and life satisfaction. Since life satisfaction is a discrete variable and also a categorical variable, ordered logistic regression is used for estimation. The model is constructed as follows:


(1) $$\mathrm{Happines}s_{i}=\alpha_{0}+\alpha_{1}\mathrm{Pensio}n_{i}+\alpha_{2}X_{i}+\varepsilon_{i}$$

In Equation (1), *Happiness_i_* represents the life satisfaction of sample *i*; $\alpha_{0}$ represents the constant term; Pensioni is the level of basic pension received by sample *i*, represented by the ratio of the local basic pension level to the minimum wage income; $X_{i}$ represents the impact of control variables on subjective well-being for sample *i*, with control variables including individual characteristic variables, number of children, social relationships, and family income; $\varepsilon_{i}$ is the random disturbance term.

Given that the degree of depression is a discrete variable, Ordinary Least Squares (OLS) regression is we used for estimation. The model is constructed as follows:


(2) $$\mathrm{Depressio}n_{i}=\beta_{0}+\beta_{1}\mathrm{Pensio}n_{i}+\beta_{2}Z_{i}+\varepsilon_{i}$$

In Equation (2), *Depression_i_* represents the degree of depression of sample *i*;

$\beta_{0}$ represents the constant term; *Pension_i_* is the level of basic pension received by sample *i*, represented by the ratio of the local basic pension level to the minimum wage income; $Z_{i}$ represents the impact of control variables on subjective well-being for sample *i*, with control variables including individual characteristic variables, number of children, social relationships, and family income; $\varepsilon_{i}$ is the random disturbance term.

### 2.2 Data

The data used in this study comes from the 2018 and 2020 panel data of the China Health and Retirement Longitudinal Study (CHARLS). The CHARLS project led by the National School of Development at Peking University, aims to collect a set of high-quality microdata to promote interdisciplinary research on aging issues. The national baseline survey of CHARLS began in 2011, and since then, the sample has been followed up and updated every 2–3 years, with new samples added to maintain its relevance. This dataset targets households and individuals aged 45 and above, covering 150 county-level units, 450 village-level units, and approximately 17,000 individuals from 10,000 households. CHARLS data extensively cover the demographic background of respondents, work, retirement and pension status, health condition, medical security, detailed family income, expenditure, asset status, etc., which is an important data source for sociology, economics, demography, and other fields. To study the impact of basic urban and rural pensions on the subjective well-being of the older adult in rural areas, this paper selected samples of rural older adult people aged 60 and above, ultimately obtaining a sample size of 9,310.

### 2.3 Explained variables

Table 1 shows the variable description. In this paper, the degree of depression and life satisfaction are used as indicators to measure the subjective well-being of the rural older adult (6, 40–43). The degree of depression is calculated based on responses to questions from the short depression scale (CES-D) in the CHARLS questionnaire. The CES-D scale is one of the best screening tools for depressive symptoms in the older adult, known for its high reliability and validity. The depression scale consisted of 10 questions related to the feelings and behaviors of the respondents in the previous week, with each question having the same set of responses: rarely or none of the time, some or a little of the time, occasionally or a moderate amount of time, and most of the time. Answers are assigned scores from 0 to 3; for questions about positive emotions, scores are assigned inversely from 3 to 0. The scores of the 10 questions are then summed and averaged to represent the degree of depression, with higher scores indicating more severe depression (6). Additionally, this paper selects life satisfaction as a positive measure of subjective well-being. The corresponding question in the CHARLS database is “Are you satisfied with your life?” Responses are scored from 1 to 5, corresponding to the options: not satisfied at all, not very satisfied, somewhat satisfied, very satisfied, and completely satisfied, with higher values indicating greater satisfaction.

**Table 1:** Variable description.

Variable properties	Variable	Variable description
Explained variables	Degree of depression	The scores for the 10 questions in the depression scale were summed and then averaged.
	Life satisfaction	Not satisfied at all = 1; Not very satisfied = 2; Somewhat satisfied = 3; Very satisfied = 4; Extremely satisfied = 5.
Explaining variables	Basic pension for urban and rural residents	Ratio of basic pension and minimum wage income for urban and rural residents.
Control variables	Age	The age of the respondent at the time of the survey.
	Gender	Male = 1; Female = 0.
	Education level	Junior high school and below =1; junior high school and above = 2.
	Marital status	With a spouse = 1; without a spouse = 0.
	Chronic disease status	Yes = 1; No = 0.
	Number of children	The number of children the respondent has.
	Personal income	The logarithm of the total wage income and transfer payment income of the respondent in the past year
	Family income	The logarithm of the respondent’s family income in the past year, including income from product sales, self-employment or private income, and public transfer payment income.
	Social relationship	Whether the respondent engaged in social activities in the past month: Yes = 1; No = 0.
Mediating variables	Health condition	Respondent’s self-rated health condition: Very bad = 1; Bad = 2; Fair = 3; Good = 4; Very good = 5
	Intergenerational support	The logarithm of the total financial support provided by children to their parents, including cash and in-kind.
	Labor supply	The number of working hours per week that the respondent worked in self-employment, employment, and as a helper in family business activities in the past year.

### 2.4 Explaining variables

This paper selects the basic pension for urban and rural residents received monthly by the sample individuals to investigate its impact on the subjective well-being of the older adult in rural areas. The data on the basic pensions for urban and rural residents in 2018 and 2020 were organized and matched with the CHARLS micro-database by province. The level of basic pension for urban and rural residents is the core explanatory variable of this paper. Due to the significant differences in the basic pension levels among some provinces, this paper uses the ratio of the basic pension for urban and rural residents to the minimum wage income to represent it.

### 2.5 Control variables

Referencing existing studies, this article includes individual characteristic variables, number of children, family income, and social relationships as control variables. The individual characteristic variables encompass age, gender, education level, marital status, chronic disease status, and personal income. The subjective well-being of the older adult is the result of the value judgment and subjective feelings of the older adult individuals on their living conditions according to their own wishes and expectations. Generally speaking, age, gender (44), education level, marital status, and other factors will have a positive impact on subjective well-being, but well-being does not necessarily increase simultaneously when income increases (45). The number of children is also an important factor that affect the subjective well-being of the older adult in rural areas, and the number of children has a significant impact on the subjective well-being of the older adult (46, 47). Children can accompany and take care of the older adult, providing spiritual comfort and support to the older adult, so parents with children have higher subjective well-being (48). From the perspective of social relations, Chinese society is a typical relationship-oriented society, “relationship” occupies an important position in the social and economic activities of residents, and plays a very important role. Compared with urban residents, rural residents pay more attention to the harmonious relationship between their neighbors. Studies have shown that social interaction has a positive impact on subjective well-being (49, 50), and the more harmonious the relationship between friends, the higher the well-being (51). Therefore, this article uses the question from the survey, “Have you engaged in the following social activities in the past month?” as a proxy variable for the respondents’ social relationships.

### 2.6 Mediating variables

Studies have shown that participation in basic pension insurance for urban and rural residents and the New Rural Pension (NRP) insurance can effectively improve the health condition of rural older adult people (52, 53), and rural older adult people have a certain degree of worry about pension issues, which is an important indicator of the quality of life for rural residents from the spiritual level, and pension income can reduce the degree of worry about the future pension, thereby improving the mental health of rural older adult people. The relationship between health conditions and subjective well-being is bidirectional. Older adult people with diseases such as coronary heart disease and chronic lung disease tend to have higher depression and impaired well-being (54). Therefore, personal health condition has a significant positive impact on subjective well-being (55), and the higher the self-rated health, the stronger the subjective well-being (56). Therefore, this article selects the self-rated health condition from the questionnaire responses to represent the physical health condition of the older adult, aiming to explore whether the basic pension for urban and rural residents affects the subjective well-being of rural older adult people through health conditions.

Additionally, considering the influence of subjective factors when respondents conduct self-rated of their health, this article also selects intergenerational support and labor supply as mediating variables. Basic pension insurance for urban and rural residents and family pension are the two primary methods of providing for the rural older adult in China, among which family pension includes intergenerational support from children to their parents. Intergenerational support refers to the financial support of children to their parents. As a son or daughter, he or she has an obligation to care for his or her parents, which includes taking care of their daily lives (57) and providing financial support, and receiving the basic pension for urban and rural residents will have a certain impact on intergenerational support. According to research, participation in pension insurance significantly increases the intergenerational economic support received by the older adult (58), and older adult people who receive support from their children have significantly higher subjective well-being than those who do not receive such support (59). Intergenerational economic support can further promote the well-being effect of pension insurance (60). Existing research proves that pension income can reduce the labor supply of the older adult to some extent (3, 60, 61). As a non-labor income, basic pension for urban and rural residents can enable the older adult to obtain more opportunities for free distribution of labor and leisure, and leisure time can allow people to meet their psychological needs such as relaxation and self-improvement and can enable rural older adult people to increase their participation in life and reduce the negative impact of aging. Since the sum of labor and leisure time is constant, when they choose to obtain leisure time, they must reduce a certain amount of labor supply. The reduction in labor participation time and the increase in leisure participation time significantly promote the subjective well-being of rural older adult people (62).

## 3 Results

### 3.1 Descriptive statistical

Table 2 reports the descriptive statistics for the variables used in this study.

**Table 2:** Descriptive statistics and description of variables.

Variable properties	Variable	Observations	Mean	Standard deviation	Minimum value	Maximum value
Explained Variables	Degree of depression	9,310	0.955	0.671	0	3
	Life satisfaction	9,310	3.288	0.791	1	5
Explaining Variables	Basic pension for urban and rural residents	9,310	0.0683	0.0163	0.0516	0.444
Control variables	Age	9,310	67.36	5.554	60	108
	Gender	9,310	0.508	0.500	0	1
	Education level	9,310	1.202	0.402	1	2
	Marital status	9,310	0.824	0.381	0	1
	Chronic disease status	9,310	0.420	0.494	0	1
	Number of children	9,310	3.098	1.441	0	13
	Personal income	9,296	6.444	3.039	0	12.49
	Family income	9,163	5.789	3.649	0	14.45
	Social relationship	9,309	0.439	0.496	0	1
Mediating variables	Health condition	9,307	2.967	1.036	1	5
	Intergenerational support	9,310	7.079	2.864	0	13.06
	Labor supply	9,310	26.60	31.96	0	320

In terms of degrees of depression, the average depression index for rural older adult in 2018 and 2020 was 0.955 points, close to the critical value of 1 point for depressive symptoms. If the older adult samples are grouped according to a depression index cutoff of 1 point, then 43.4% of the rural older adult with a depression index of 1 point and above show symptoms of mental depression. The average life satisfaction score for rural older adult is 3.288, indicating that overall, they are “relatively satisfied” with their lives. The basic pension indicator for urban and rural residents is the ratio of the basic pension for urban and rural residents to the minimum wage income. The average level of basic pension for urban and rural residents in the sample overall is 0.0683, the minimum value in the sample is 0.0516, and the maximum value is 0.444. The mean value is closer to the minimum value in the sample, reflecting that the current basic pension for urban and rural residents in China is still at a low level, with only a few regions having basic pension levels much higher than the minimum standard.

### 3.2 The estimation results of the impact of rural and urban residents’ basic pension on the subjective well-being of rural older adult

Table 3 presents the OLS regression estimates for the impact of basic pension levels on the degrees of depression among the older adult in rural areas and the ordered logit regression estimates for life satisfaction. Column (1) shows the univariate OLS regression results, indicating a significant negative impact of basic pension levels on the degrees of depression among rural older adult, indicating that higher levels of basic pensions are associated with lower degrees of depression and higher subjective well-being. After adding control variables into the regression, the results in column (2) still show a significant negative effect of basic pension levels on depression. Similarly, column (3) presents the univariate ordered logit regression results for the impact of basic pension levels on life satisfaction, revealing a significant positive effect, meaning that higher basic pension levels are associated with higher life satisfaction and greater subjective well-being among rural older adult. After the introduction of control variables, the results in column (4) remain significant. These findings indicate that basic pension levels have a significant positive impact on the subjective well-being of the older adult in rural areas, regardless of whether the measure is a positive or negative indicator of subjective well-being. Most of the older adult living in rural China do not have neither fixed jobs nor stable incomes. With the growth of age, their working ability gradually declines, and their means of obtaining income gradually decrease. However, the basic pension income of urban and rural residents, although not high in amount, alleviates the pressure of insufficient economic sources for the older adult in rural areas. Through this pension, the consumption expenditure of durable goods and the expenditure of medical care can be improved effectively, so as to improve the quality of life of the rural older adult and improve their life satisfaction. In addition, continuous and stable access to pensions will also make the rural older adult feel the stability of life and the security of the future, thus increasing their subjective sense of well-being.

**Table 3:** Basic pension level and subjective well-being of rural older adult.

	Degrees of depression	Degrees of depression	Life satisfaction	Life satisfaction
	(1)	(2)	(3)	(4)
Basic pension for urban and rural residents	−2.224^***^ (0.426)	−1.727^***^ (0.416)	4.206^***^ (1.219)	3.652^***^ (1.228)
Age		−0.004^***^ (0.001)		0.020^***^ (0.004)
Gender		−0.194^***^ (0.014)		−0.005 (0.042)
Education level		−0.140^***^ (0.018)		−0.158^***^ (0.052)
Marital status		−0.165^***^ (0.018)		0.078 (0.056)
Chronic disease status		0.165^***^ (0.014)		−0.224^***^ (0.041)
Number of children		0.027^***^ (0.005)		−0.011 (0.016)
Personal income		−0.016^***^ (0.002)		0.027^***^ (0.007)
Family income		0.007^***^ (0.002)		−0.002 (0.006)
Social relationship		−0.069^***^ (0.014)		0.089^**^ (0.040)
Sample size	9,310	9,148	9,310	9,148
*R* ^2^	0.003	0.084	0.001	0.005

^*^, ^**^, and ^***^ indicate significance level at 1%, 5%, and10%, respectively. Standard errors in brackets.

In column (2), regarding the estimation results for control variables, all included control variables show a significant correlation with the subjective well-being of the older adult in rural areas. Age, gender, level of education, having a spouse, personal income, and social relationships all have a significant positive effect on the subjective well-being of rural older adult. Age is significant at the 1% level, indicating that older rural older adult experience lower degrees of depression and higher levels of subjective well-being. The possible explanation is that with the increase of age, the life pressure of the rural older adult is gradually alleviated, and they have more time to pay attention to their own feelings and needs, so they have better psychological state and higher level of subjective well-being; the level of subjective well-being in male groups is higher than that in female groups; rural older adult with higher education levels have a higher level of well-being, possibly because different educational background will have different degrees of impact on individual income and social status; rural older adult with spouses have higher levels of subjective well-being than those without spouses. The reason for this could be that rural older adult with spouses can receive emotional support and care from their spouses, thereby reducing depression and enhancing well-being; the higher the level of personal income, the higher the level of well-being, and high-income levels can bring good material living conditions, and bring economic security and stability, which can reduce the economic pressure and improve the level of well-being; rural older adult social activities can significantly increase the level of subjective well-being, and social interaction activities can help strengthen emotional communication, provide spiritual support and emotional satisfaction for the rural older adult, reduce the degree of mental depression, and enhance the sense of pleasure and happiness.

Having chronic diseases, the number of children, and family income have a significant negative impact on the subjective well-being of rural older adult. The subjective well-being level of the rural older adult with chronic diseases is lower than that of the rural older adult without chronic diseases. Chronic diseases bring both mental and physical burdens to the rural older adult. Most of the people with chronic diseases need long-term medication or treatment, and the expenditure on medical expenses will bring economic pressure to the older adult, which may make them feel anxious and uneasy, increase the probability of falling into health poverty, and thus reduce the level of subjective well-being; the more the number of children, the lower the subjective well-being, which may be due to the fact that the children cannot go home often for migrant work, lack of communication with their parents, or the family relationship is not harmonious, so the increase in the number of children will reduce the subjective well-being of the older adult in the rural areas. From the perspective of income, a higher personal income level can enhance the level of well-being, whereas a higher family income level tends to reduce well-being. This paradoxical effect may suggest that while personal income contributes directly to an individual’s sense of security and ability to fulfill personal desires, higher household income could potentially lead to increased responsibilities, expectations, or stress within the family context, thereby negatively affecting the individual’s subjective well-being.

### 3.3 Robustness check

#### 3.3.1 Replacement regression model

Poisson regression is a generalized linear model. Poisson regression assumes that the conditional distribution of the explained variable is the Poisson distribution, and predicts the expected value of the explained variable by introducing the explaining variable. Considering that different regression models may affect the results of hypothesis testing, this study further uses Poisson regression model to regress the data (63) to verify the impact of the basic pension of urban and rural residents on the subjective well-being of the rural older adult in China. Table 4 presents the results of the Poisson regression. The stability of the regression coefficient and the regression results in Table 4 show a high consistency with those in Table 3. Both before and after the inclusion of control variables, the level of basic pension has a significant negative impact on the degrees of depression among the older adult in rural areas. That is, in areas with higher basic pension levels, the degrees of depression among rural older adult are lower, and their subjective well-being is higher. This confirms the robustness of the research conclusions stated above.

**Table 4:** Poisson regression results.

	Degrees of depression	Degrees of depression
	(1)	(2)
Basic pension for urban and rural residents	−2.661^***^ (0.535)	−2.074^***^ (0.484)
Age		−0.004^***^ (0.001)
Gender		−0.204^***^ (0.015)
Education level		−0.165^***^ (0.020)
Marital status		−0.159^***^ (0.018)
Chronic disease status		0.171^***^ (0.014)
Number of children		0.027^***^ (0.005)
Personal income		−0.017^***^ (0.002)
Family income		0.008^***^ (0.002)
Social relationship		−0.072^***^ (0.014)
Constant	0.135^***^ (0.037)	0.748^***^ (0.110)
Sample size	9,310	9,148
*R* ^2^	0.001	0.018

Standard errors in parentheses ^*^p < 0.1, ^**^p < 0.05, and ^***^p < 0.01.

#### 3.3.2 Replacing the explained variable

The method of replacing the explained variables is to use alternative variables that are highly correlated with the original variables but have different data sources or different calculation methods and re-run the regression analysis to test the robustness of the regression results.

With the migration of rural labor force, the miniaturization and decentralization of rural family structure, and the lack of older adult care have brought many psychological and emotional problems to the older adult, and a large number of rural older adult have been attacked by loneliness. Research has found that loneliness and life satisfaction in the older adult are significantly negatively correlated. Higher levels of loneliness tend to lead to a variety of negative emotional experiences, which in turn reduce levels of life satisfaction (64). If the basic pension for urban and rural residents can reduce the loneliness of the older adult, it will reduce the negative emotions of the rural older adult, and thus enhance the subjective well-being of the rural older adult. Therefore, this article uses the “loneliness” variable as a proxy variable for the subjective well-being of the rural older adult to regress. According to the question “I feel lonely” in the 2018 and 2020 CHARLS questionnaires, the questionnaires classify loneliness as a quadratic variable: “Rarely or not at all (<1 day),” “not much (1–2 days),” “sometimes or half of the time (3–4 days),” “most of the time (5–7 days),” and re-assigned a score of 1–4, with higher scores indicating stronger feelings of loneliness. In this way, it is investigated whether the basic pension for urban and rural residents will similarly affect the loneliness of the rural older adult, thus confirming the robustness of the research results. Table 5 reports the regression results focusing on loneliness, and columns (1) and (2) adopt the ordered logistic estimation method. The regression results show that the basic pension for urban and rural residents has a significant negative impact on the loneliness of rural older adult. The results using loneliness as the explained variable are similar to the empirical results of depression degree, indicating that the above analysis results are relatively robust. It further explains that the basic pension can provide spiritual comfort and support to the rural older adult, reduce their loneliness, and enhance their level of subjective well-being.

**Table 5:** Regression results replacing the explained variable.

	孤独感	孤独感
	(1)	(2)
Basic pension for urban and rural residents	−4.917^***^ (1.509)	−4.150^***^ (1.547)
Age		−0.006 (0.005)
Gender		−0.202^***^ (0.047)
Education level		−0.164^***^ (0.060)
Marital status		−1.104^***^ (0.056)
Chronic disease status		0.338^***^ (0.045)
Number of children		0.038^**^ (0.017)
Personal income		−0.045^***^ (0.007)
Family income		0.014^**^ (0.006)
Social relationship		−0.127^***^ (0.045)
Sample size	9,310	9,148
*R* ^2^	0.001	0.033

Standard errors in parentheses ^*^p < 0.1, ^**^p < 0.05, and ^***^p < 0.01.

### 3.4 Heterogeneity analysis

The above analysis results show the impact of the basic pension for urban and rural residents on the subjective well-being of the rural older adult. However, this impact is based on the sample model analysis and does not take into account the differences between the gender, health condition, and regions of the rural older adult.

Since the differences between male and female in mental sensitivity may affect the cognitive experience and emotional circuit of subjective well-being, grouping the samples according to gender ratio can reflect the difference in the impact of the basic pension for urban and rural residents on the subjective well-being of male and female; The payment of basic pension for urban and rural residents can produce health effects. For older adult people without chronic diseases, sustained and stable basic pension income can reduce individual depression, while the older adult with chronic diseases face medical expenses, so they have a higher dependence on basic pension and greater life security brought by basic pension, thus improving their subjective well-being. They are grouped according to whether they suffer from chronic diseases to explore the different impacts of basic pension on rural older adult with different health conditions; Under China’s unique dual economic structure, there is still a big gap in the level of economic development among different regions, and there are differences in social system, development situation, external environment, and other aspects in different regions. Therefore, under different constraints, the acceptance and expectation of the basic pension policy for urban and rural residents in different regions are different. Therefore, the differences in the subjective well-being of the rural older adult in different regions can be reflected by the grouping of eastern, central, and western regions. Therefore, grouping according to the eastern, central, and western regions can reflect the differences in subjective well-being among rural older adult in different regions.

Therefore, this article conducts group regression on the entire sample from three perspectives: gender ratio of rural older adult, whether they suffer from chronic diseases, and eastern, central and western regions, to examine the heterogeneous impact of basic pension for urban and rural residents on the subjective well-being of rural older adult. The specific results are shown in Table 6.

**Table 6:** Heterogeneity analysis of basic pension levels and degrees of depression among rural older adult.

Variable	Degrees of depression						
	Gender ratio		Chronic disease status		Region		
	(1) High category	(2) Low category	(3) Suffering from chronic diseases	(4) Not suffering from chronic diseases	(5) Eastern region	(6) Central region	(7) Western Region
Basic pension for urban and rural residents	−2.336^***^ (0.704)	−1.279^**^ (0.518)	−2.064^***^ (0.621)	−1.426^**^ (0.564)	−1.956^***^ (0.526)	14.252^***^ (1.895)	4.273^***^ (0.976)
Age	−0.006^***^ (0.002)	−0.003 (0.002)	−0.004^*^ (0.002)	−0.004^***^ (0.002)	−0.006^***^ (0.002)	−0.000 (0.003)	−0.005^*^ (0.003)
Gender	−0.170^***^ (0.020)	−0.213^***^ (0.020)	−0.243^***^ (0.023)	−0.160^***^ (0.018)	−0.171^***^ (0.022)	−0.198^***^ (0.025)	−0.230^***^ (0.026)
Education level	−0.095^***^ (0.025)	−0.185^***^ (0.025)	−0.122^***^ (0.029)	−0.152^***^ (0.022)	−0.118^***^ (0.027)	−0.098^***^ (0.029)	−0.167^***^ (0.037)
Marital status	−0.187^***^ (0.027)	−0.145^***^ (0.025)	−0.155^***^ (0.029)	−0.172^***^ (0.024)	−0.174^***^ (0.030)	−0.194^***^ (0.031)	−0.078^**^ (0.034)
Chronic disease status	0.160^***^ (0.020)	0.166^***^ (0.019)	0.000 (.)	0.000 (.)	0.150^***^ (0.022)	0.192^***^ (0.023)	0.138^***^ (0.025)
Number of children	0.038^***^ (0.008)	0.018^***^ (0.007)	0.025^***^ (0.008)	0.029^***^ (0.007)	0.015^*^ (0.009)	0.023^**^ (0.010)	0.008 (0.009)
Personal income	−0.016^***^ (0.003)	−0.016^***^ (0.003)	−0.017^***^ (0.004)	−0.016^***^ (0.003)	−0.019^***^ (0.003)	−0.019^***^ (0.004)	−0.006 (0.004)
Family income	0.009^***^ (0.003)	0.005^**^ (0.003)	0.011^***^ (0.003)	0.005^**^ (0.002)	0.005^*^ (0.003)	0.004 (0.003)	−0.005 (0.004)
Social relationship	−0.074^***^ (0.020)	−0.063^***^ (0.019)	−0.095^***^ (0.022)	−0.049^***^ (0.017)	−0.049^**^ (0.021)	−0.076^***^ (0.023)	−0.057^**^ (0.026)
Sample size	4,437	4,711	3,845	5,303	3,320	2,964	2,864
*R* ^2^	0.079	0.090	0.073	0.064	0.083	0.108	0.069

The estimation results indicate that, from the perspective of gender ratio grouping, the impact of basic pension is greater in provinces with a relatively higher gender ratio, in terms of the absolute value of the coefficient. The gender ratio is the proportion of males to females within a province, with a higher ratio indicating a higher proportion of males in the sample of that province. Therefore, the impact of the basic pension on the subjective well-being of males is greater than females. This might be because men typically bear the responsibility of being the primary source of income in the family, thereby facing greater psychological stress. Consequently, basic pension income can alleviate some of the burden on the male population to a certain extent, enhancing their subjective well-being.

From the perspective of whether individuals have chronic diseases, the level of basic pension for urban and rural residents has a significantly positive effect on the subjective well-being of both groups of samples, with significance at the 1% level. The gap in significance between the two groups is not large, but the absolute value of the regression coefficient for the sample with chronic diseases is slightly higher than that for the sample without chronic diseases. The reason is that for rural older adult people suffering from chronic diseases, the income from the basic pension for urban and rural residents can offset part of their medical expenses, thereby reducing their living burden and improving their subjective well-being.

Looking at the regional grouping, the level of basic pension for urban and rural residents has a significant negative impact on the degrees of depression of rural older adult in the eastern region, and is significant at the 1% significance level, indicating that the basic pension for urban and rural residents can improve the level of subjective well-being among rural older adult in the eastern region. The possible explanation is that the economy in the eastern region is generally more developed, the social security system is relatively complete, the level of basic pension benefits is relatively high, and residents have high incomes, and thus the pressure of contribution is small, and the rural older adult in this region are more likely to improve their quality of life, reduce the level of depression, and enhance their subjective well-being through the basic pension. In addition, the regression results show that the level of basic pension has a significant positive effect on the depression degree of rural older adult in the central and western regions, indicating that the basic pension suppresses the subjective well-being of rural older adult in the central and western regions. The possible reason is that the level of economic development in the central and western regions lags behind, and the living standards of the rural older adult are poorer. Basic pension can improve their living standards to a certain extent, but the lower level of pension benefits is not enough to meet their living needs, which may lead to an increase in the degree of depression. The impact of the pension level on the eastern and central regions is more significant than that on the western region, probably because of the development level and cultural differences between regions. The eastern region is more economically developed, cultural exchanges are more active, and the ideology of the rural older adult is more open, and the impact of the pension level is more significant. However, the rural older adult in the western region have relatively conservative ideas and basic pension levels have relatively little impact on them. Among these three sample groups, the absolute value of the regression coefficient for the central region is the largest. The possible reason is the difference in the basic pension levels among regions, which is indeed the case, with the basic pension level in the eastern region being significantly higher than in the central and western regions.

### 3.5 Mechanism of influence

This study employs the mediation effect method to test whether the basic pension affects the subjective well-being of the rural older adult through three factors: health condition, intergenerational support, and labor supply. The mediation effect model studies how the independent variables affect the dependent variable by analyzing the path of the independent variables on the dependent variable. In empirical analysis, the commonly used approach for the mediation effect test is the stepwise regression method (65–68), which is to determine whether the mediation effect exists or not by multiple regressions, based on the significance of the regression coefficients. The mediation effect model used in this article is constructed as follows:


(3) $$\mathrm{Depressio}n_{i}=\theta_{1}+\mathrm{cPensio}n_{i}+\gamma CV_{i}+\varepsilon_{i}$$

(4) $$M_{i}=\theta_{2}+\mathrm{aPensio}n_{i}+\gamma CV_{i}+\varepsilon_{i}$$

(5) $$\mathrm{Depressio}n_{i}=\theta_{3}+c^{\prime }\mathrm{Pensio}n_{i}+bM_{i}+\gamma CV_{i}+\varepsilon_{i}$$

In Equations (3–5), *a*, *b*, *c*, *c′* are regression coefficients, $\mathrm{Depressio}n_{i}$ representing the degree of depression of the explained variable; $\mathrm{Pensio}n_{i}$ representing the basic pension level of the explaining variable; $M_{i}$ representing mediating variable, including health condition, intergenerational support and labor supply; $CV_{i}$ and are control variables. According to the mediation effect test process of previous studies, the first step is to test whether the influence coefficient *c* of the basic pension level on the depression degree of rural older adult is significant, and the second step is to test whether the influence coefficient *a* of the basic pension level on the mediating variable is significant. If the two coefficients are significant, then test whether coefficient *b* is significant. If it is significant, it indicates that there is a mediating effect. If one of *a* and *c* is not significant, use the Bootstrap method to test $H_{0}:\mathrm{ab}=0$ . If it is significant, there is a mediating effect. If it is not significant, there is no mediating effect. Table 7 presents the analysis of the mechanism through which the level of basic pension influences the degrees of depression among rural older adult individuals.

**Table 7:** Analysis of the impact mechanism of basic pension level on the degrees of depression.

Variable	Degrees of depression	Health condition		Intergenerational support		Labor supply	
		Health condition	Degrees of depression	Financial support	Degrees of depression	Working hours	Degrees of depression
	(1)	(2)	(3)	(4)	(5)	(6)	(7)
Basic pension for urban and rural residents	−1.727^***^ (0.416)	2.973^***^ (0.646)	−1.056^***^ (0.390)	2.674 (1.773)	−1.697^***^ (0.416)	−89.837^***^ (19.373)	−1.786^***^ (0.416)
Health condition			−0.225^***^ (0.006)				
Financial support					−0.011^***^ (0.002)		
Working hours							−0.001^***^ (0.000)
Control variables	Control	Control	Control	Control	Control	Control	Control
Sample size	9,148	9,145	9,145	9,148	9,148	9,148	9,148
*R* ^2^	0.084	0.072	0.195	0.088	0.086	0.124	0.084

Financial support refers to the logarithm of financial support from children to their parents.

Columns (2, 3, 6, 7) show that the coefficients have passed the tests, indicating that health conditions and labor supply have mediating effects in the impact of basic pension on the degrees of depression of rural older adult individuals; whereas column (4) shows that the coefficient for intergenerational support is not significant and does not pass the Bootstrap test, indicating the absence of a mediating effect of intergenerational support. This study empirically tests and verifies the mediating roles of health status and labor supply.

Based on the above analysis, it is evident that due to the older adult being reluctant to increase the burden of their children or the relative backwardness of rural medical resources, most older adult individuals do not seek treatment immediately after falling ill. However, rural older adult individuals use their basic pension for medical expenses, thereby improving their health condition and enhancing their subjective well-being. Moreover, for most rural older adult individuals, if there is no income from the basic pension, who would need to engage in more labor to earn income to sustain their lives in old age. A fixed basic pension income can provide some material security for the older adult, reduce their psychological burden, and release them from heavy labor activities, thereby enhancing their subjective well-being.

## 4 Discussion

In recent years, with the development of China’s economy and the improvement of people’s living standards, residents’ demands for quality of life have shifted from material aspects such as income and consumption to a greater emphasis on spiritual well-being. Research has found that individuals are able to explicitly evaluate their happiness in terms of overall well-being, meaning, and psychological richness (69). This paper constructs OLS and ordered logistic models and uses data from the 2018 and 2020 China Health and Retirement Longitudinal Study to perform regression analysis, estimating the impact of the basic pension for urban and rural residents on the subjective well-being of the rural older adult. The regression results show that the basic pension for urban and rural residents significantly enhances the level of subjective well-being among the rural older adult. Further evidence indicates that the impact of the basic pension on the subjective well-being of men is greater than that of women. The basic pension has a significant positive impact on the subjective well-being of the rural older adult with chronic diseases. The level of the basic pension has a significant negative impact on the depression levels of the rural older adult in the eastern regions. The analysis of the mechanism of impact based on the mediating effect method shows that health conditions and labor duration play an important role in the process of the impact of basic pension for urban and rural residents on subjective well-being. Improving health conditions and reducing labor supply can enhance the subjective well-being of the rural older adult.

Based on the findings of this study, the policy implications of this paper are as follows:

On the one hand, enhance the level of basic pension benefits for urban and rural residents based on policy goals. Due to the differences in economic development level and fiscal subsidy capacity among different regions, the development of basic pension insurance for urban and rural residents in different regions is not balanced, especially the basic pension in central and western regions is generally low, which is not only the reason of system design, but also the result of local financial capacity. Therefore, it is recommended that the central government increase financial subsidies, improve basic pension benefits for urban and rural residents, meet the basic living needs of the rural older adult, and effectively improve the subjective feelings of the rural older adult.

On the other hand, the basic pension should be optimized and upgraded to a non-contributory pension in terms of system practice. Academic research has proven that pensions obtained through social security have a strong positive impact on personal happiness (70). For the rural older adult, due to the lack of pension accumulation during employment, the most effective and direct way is to obtain a “non-contributory pension” with inclusive nature. The basic pension for urban and rural residents is very similar to the non-contributory pension, which is a kind of “non-contributory pension” in a sense. Because the basic pension for urban and rural residents does not have any accumulation of funds, it is completely from fiscal transfer payments, and has nothing to do with personal contributions, which reflects the non-contributory and welfare nature of the system. Some studies indicate that non-contributory pension schemes targeting the poor population in developing countries can improve the welfare of the poor older adult (71), and non-contributory social pensions can effectively reduce the incidence of older adult poverty and extreme poverty, playing a positive role in poverty reduction (72). Therefore, optimizing and upgrading the basic pension for urban and rural residents into a non-contributory pension can more effectively solve the problem of low basic pension benefits, rapidly expand the coverage of pension insurance, and eliminate poverty in the older adult.

Finally, it needs to be discussed that the relevant welfare policies of the basic pension insurance for urban and rural residents should be tilted toward vulnerable groups such as rural female and left-behind older adult people. Although the implementation of the policy of focusing on the key groups will face many difficulties in practice and will greatly increase administrative cost, the policy effect is obvious. The regression results indicate that the improvement of income level can significantly improve the well-being of rural female older adult and left-behind older adult. Therefore, the government can provide multi-level protection for this low-income group in the form of strengthening local responsibilities. For example, in addition to the basic pension provided by the central government, a certain number of pension subsidies provided by the local government can be added, so as to compensate the income gap of vulnerable groups in society and improve the well-being of vulnerable older adult groups.

This study has several limitations. Firstly, the research data used only consists of two periods of panel data, which only explores the relationship between the current basic pension for urban and rural residents and the subjective well-being of rural older adult. However, the level of the basic pension for urban and rural residents may be adjusted in later periods. Therefore, the relationship between the basic pension level for urban and rural residents in the same region and the well-being of rural older adult requires further exploration. Secondly, although the article controls for some variables during the empirical process, subjective well-being is influenced by a variety of factors, and there may still be issues with the selection of indicators not being comprehensive enough.

## Data availability statement

The original contributions presented in the study are included in the article/supplementary material, further inquiries can be directed to the corresponding author.

## Ethics statement

Ethical approval was not required for the study involving humans in accordance with the local legislation and institutional requirements. Written informed consent to participate in this study was not required from the participants or the participants’ legal guardians/next of kin in accordance with the national legislation and the institutional requirements.

## Author contributions

JY: Writing – original draft, Conceptualization, Formal analysis, Methodology. ZL: Software, Writing – original draft. JZ: Data curation, Writing – original draft. ZZ: Funding acquisition, Project administration, Supervision, Writing – review & editing.

## References

[1] Mu G. Reform and prospect of traditional pension plan for the aged in China. J Renmin Univ Chin. (2000) 5:39–44.

[2] Shu F. From family supporting to social pension: a study on the changes of rural old-age Care in the Past 70 years in new China. Zhejiang Soc Sci. (2019) 6:83–91. doi: 10.14167/j.zjss.2019.06.009

[3] Zhang C Giles J Zhao Y. A policy evaluation of China's new rural pension Programme: income, poverty, expenditure, subjective well-being and labour supply. Chin Econ Q. (2014) 14:203–30. doi: 10.13821/j.cnki.ceq.2015.01.012

[4] He H Li X. Evaluation of the impact of the new rural social pension insurance policy on rural residents’ consumption. J Jiangxi Univ Fin Economics. (2020) 3:61–72. doi: 10.13676/j.cnki.cn36-1224/f.2020.03.006

[5] Zhu H. Assessing the poverty reduction effect of the urban and rural resident pension: from the perspective of multidimensional poverty. Soc Sci Beijing. (2017) 9:112–9. doi: 10.13262/j.bjsshkxy.bjshkx.170912

[6] He Y Zhou Q. A study on the effect of Chinese new rural pension program on rural subjective well-being. Insur Stud. (2016) 3:106–17. doi: 10.13497/j.cnki.is.2016.03.010

[7] Wang Y Long Y Jiang C Xu Q. Research on social security income redistribution effect in China. Econ Res J. (2016) 2:4–15.

[8] Ma H Xi H. Income gap, social insurance and improvement of residents' happiness. Soc Sec Stud. (2020) 1:86–98. doi: 10.3969/j.issn.1674-4802.2020.01.009

[9] Diener E Ryan K. Subjective well-being: a general overview. S Afr J Psychol. (2009) 39:391–406. doi: 10.1177/008124630903900402, PMID: 38632112

[10] Pak TY. Social protection for happiness? The impact of social pension reform on subjective well-being of the Korean older adult. J Policy Model. (2020) 42:349–66. doi: 10.1016/j.jpolmod.2019.12.001

[11] Chen Y Tan Y (2017). Income and subjective well-being: evidence from Singapore's first national non-contributory pension. CREA Working Paper.

[12] Zhong C Yue X. Does economic growth improve human happiness? A review of the influencing factors of SWB. Nankai Econ Stud. (2020) 4:24–45. doi: 10.14116/j.nkes.2020.04.002

[13] Blanchflower DG Oswald AJ. Well-being over time in Britain and the USA. J Public Econ. (2004) 88:1359–86. doi: 10.1016/S0047-2727(02)00168-8, PMID: 38492116

[14] Deaton A Stone AA. Understanding context effects for a measure of life evaluation: how responses matter. Oxf Econ Pap. (2016) 68:861–70. doi: 10.1093/oep/gpw022, PMID: 29129941 PMC5679282

[15] Schatz E Gómez-Olivé X Ralston M Menken J Tollman S. The impact of pensions on health and wellbeing in rural South Africa: does gender matter? Soc Sci Med. (2012) 75:1864–73. doi: 10.1016/j.socscimed.2012.07.004, PMID: 22884944 PMC3475956

[16] Horley J Lavery JJ. Subjective well-being and age. Soc Indic Res. (1995) 34:275–82. doi: 10.1007/BF01079200, PMID: 38700724

[17] MacKerron G. Happiness economics from 35 000 feet. J Econ Surv. (2012) 26:705–35. doi: 10.1111/j.1467-6419.2010.00672.x

[18] Cetre S Clark AE Senik C. Happy people have children: choice and self-selection into parenthood. Eur J Popul. (2016) 32:445–73. doi: 10.1007/s10680-016-9389-x, PMID: 28713186 PMC5505668

[19] Mendiratta A Jain A Malhotra K. Resilience and life satisfaction: predictors of well-being in pensioners and non-pensioners. Int J Ind Psychȯl. (2022) 10:446–52. doi: 10.25215/1002.045

[20] Bonsang E Klein TJ. Retirement and subjective well-being. J Econ Behav Organ. (2012) 83:311–29. doi: 10.1016/j.jebo.2012.06.002, PMID: 38696237

[21] Baker M Gruber J Milligan KS (2009). Retirement income security and well-being in Canada. NBER Working Paper No.14667.

[22] Etinzock MN Kollamparambil U. Subjective well-being impact of old age pension in South Africa: a difference in difference analysis across the gender divide. S Afr J Econ Manag Sci. (2019) 22:1–12. doi: 10.4102/sajems.v22i1.2996

[23] Etinzock MN (2018). Happiness and the older adult: a subjective wellbeing impact of social grants in South Africa. University of the Witwatersrand, Faculty of Law, Commerce and Management, School of Economics and Finance. Available at: https://wiredspace.wits.ac.za/server/api/core/bitstreams/1c73eb96-9772-4a1b-aa36-bbec86f6072e/content

[24] Luo C. Urban-rural divide, employment, and subjective well-being. Chin Econ Q. (2006) 2:817–40.

[25] He L Pan C. Uncover the “Easterlin paradox” of China: income gap, inequality of opportunity and happiness. Manag World. (2011) 8:11–22. doi: 10.19744/j.cnki.11-1235/f.2011.08.003

[26] Yang J Zhang Y. Pricing air pollution: an analysis based on happiness data. J World Econ. (2014) 12:162–88. doi: 10.19985/j.cnki.cassjwe.2014.12.009

[27] Lamu AN Olsen JA. The relative importance of health, income and social relations for subjective well-being: an integrative analysis. Soc Sci Med. (2016) 152:176–85. doi: 10.1016/j.socscimed.2016.01.046, PMID: 26854627

[28] Liu Y. Empirical study on the happiness effect of social security systems: a perspective based on medical insurance and pension insurance. J Commer Econ. (2015) 6:92–4. doi: 10.3969/j.issn.1002-5863.2015.06.036

[29] Deng D Yang J. Pension insurance, consumption inequality and subjective happiness of rural elderly: an empirical analysis using the China household financial survey data. Chin J Popul Sci. (2019) 4:43–55.

[30] Bando R Galiani S Gertler P (2017). The effects of noncontributory pensions on material and subjective well-being. IDB Working Paper Series No. IDB-WP-840.

[31] Lloyd-Sherlock P Barrientos A Moller V Saboia J. Pensions, poverty and wellbeing in later life: comparative research from South Africa and Brazil. J Aging Stud. (2012) 26:243–52. doi: 10.1016/j.jaging.2012.02.003

[32] Olivera J Ponomarenko V. Pension insecurity and wellbeing in Europe. J Soc Policy. (2017) 46:517–42. doi: 10.1017/S0047279416000787, PMID: 22786921

[33] Shao C Li W. Pension level, subjective wellbeing, and preference of care model among elderly people: an empirical study based on structural equation modeling. Front Public Health. (2023) 11:1104556. doi: 10.3389/FPUBH.2023.1104556, PMID: 36844815 PMC9947843

[34] Danzer AM Danzer N (2016). Pension generosity and mental wellbeing: The effect of eradicating poverty at old-age. Paper presented at Demographischer Wandel-session: pensions and labor market outcomes conference, No. G05-V1, Germany.

[35] Han J Zhang X Meng Y. The impact of old-age pensions on the happiness level of elderly people–evidence from China. Ageing Soc. (2022) 42:1079–99. doi: 10.1017/S0144686X20001452

[36] Li H School B, University F. Review and evaluation on rural-urban residents social pension insurance: based on replacement rate of basic pension insurance. Popul Econ. (2015) 5:91–9. doi: 10.3969/j.issn.1000-4149.2015.05.010

[37] Xu T Wei Y. Adjustment mechanism and calculation of basic pension benefits of urban and rural Residents' pension insurance under the background of rural revitalization. J Fujian Norm Univ. (2021) 6:98–109. doi: 10.12046/j.issn.1000-5285.2021.06.010

[38] Wang Z. The target treatment and level of measurement about basic pension of urban and rural residents: take six provinces in the east, middle and west of China as an example. Statist Inform Forum. (2020) 11:93–102. doi: 10.3969/j.issn.1007-3116.2020.11.011

[39] Zhao Y Zhou J. Does new rural social endowment insurance improve the happiness of middle aged and elderly people: an empirical study based on China family panel studies data. Chin Econ Stud. (2021) 6:105–22. doi: 10.19365/j.issn1000-4181.2021.06.08

[40] Zheng X Fang X. The social pension scheme and the subjective well-being of the elderly in rural China. J Financ Econ. (2018) 9:80–94. doi: 10.16538/j.cnki.jfe.2018.09.005

[41] Ni Z Li Q. Does old-age security enhance residents’ subjective well-being? Mod Econ Res. (2021) 10:31–40. doi: 10.13891/j.cnki.mer.2021.10.005

[42] Chen F Liu J Liu X. The influencing mechanism of ‘new rural social pension policy’on subjective well-being of the elderly in rural China. Res Fin Econ Issues. (2020) 1:111–20. doi: 10.19654/j.cnki.cjwtyj.2020.01.013

[43] Alzua ML Cantet MN Dammert AC Olajide D (2023). The wellbeing effects of an old age pension: experimental evidence for Ekiti state in Nigeria. Documento de Trabajo Nro 322.

[44] Alesina AF Di Tella R Macculloch R. Inequality and happiness: are Europeans and Americans different? Soc Sci Electron Publish. (2004) 88:2009–42. doi: 10.2139/ssrn.293781

[45] Easterlin RA McVey LA Switek M Sawangfa O Zweig JS. The happiness-income paradox revisited. Proc Natl Acad Sci USA. (2010) 107:22463–8. doi: 10.1073/pnas.1015962107, PMID: 21149705 PMC3012515

[46] Yu X Chen Y Qu X Huang X. Related factors on subjective well-being in the elderly. Chin Ment Health J. (2016) 6:427–34. doi: 10.3969/j.issn.1000-6729.2016.06.006

[47] Gao Y Yang Q. The current situation and main influencing factors of the happiness level of elderly people receiving home-based care. Chin J Gerontol. (2020) 17:3771–3. doi: 10.3969/j.issn.1005-9202.2020.17.057

[48] Stutzer A Frey BS. Does marriage make people happy, or do happy people get married? J Socio Econ. (2004) 35:326–47. doi: 10.2139/ssrn.375960

[49] Aslam A Corrado L (2007). No man is an island, the inter-personal determinants of regional well-being in Europe. Cambridge Working Papers in Economics 0717.

[50] Portela M Neira I Salinas-Jiménez MDM. Social capital and subjective wellbeing inEurope: a new approach on social capital. Soc Indic Res. (2013) 114:493–511. doi: 10.1007/s11205-012-0158-x

[51] Knight J Gunatilaka R. Does economic growth raise happiness in China? Oxf Dev Stud. (2011) 39:1–24. doi: 10.1080/13600818.2010.551006

[52] Kuang M He F. Study on the impact of the ‘new rural social pension policy’ on the health of the rural elderly population and the optimization of elderly care services. Rural Economy. (2018) 10:84–90.

[53] Wu Y Hu J Nie J. Study on the impact mechanism of the basic pension Insurance for Urban and Rural Residents on the health performance of the rural elderly. Soc Secur Stud. (2021) 6:10–22. doi: 10.3969/j.issn.1674-4802.2021.06.002

[54] Steptoe A Deaton A Stone A. Subjective wellbeing, health and ageing. Lancet. (2015) 385:640–8. doi: 10.1016/S0140-6736(13)61489-0, PMID: 25468152 PMC4339610

[55] Huang Y Li Y Clark WAV. Family connections and subjective wellbeing in transitional China. J Happiness Stud. (2024) 25:33. doi: 10.1007/s10902-024-00744-9

[56] Wang S. Analysis and evaluation of the structure of subjective well-being of Chinese residents——an empirical study based on CGSS2013 data. Mod Econ Sci. (2018) 3:66–74.

[57] Zang Z. The care types choice in filial culture: a cross-sectional study of disabled elderly in China. Front Public Health. (2022) 10:954035. doi: 10.3389/fpubh.2022.954035, PMID: 36148366 PMC9485573

[58] Liu P Sun L. The impact of endowment Insurance for Urban and Rural Residents on intergenerational economic support: research based on the mediating effect model. J Yunnan Fin Trade Instit. (2020) 12:3–18. doi: 10.16537/j.cnki.jynufe.000649

[59] Wang J Xu Q. Living arrangement, intergenerational support, and the subject well-being of the olders in China. J Soc Econ Dev. (2020) 7:193–208.

[60] Sun Z Zhao Q Zhao Y. Does the “new-type pension insurance for rural residents” really reduce the labor participation of the rural elderly? A study based on cross-sectional data of two periods of CHARLS. Commer Res. (2020) 10:117–26. doi: 10.13902/j.cnki.syyj.2020.10.012

[61] Zhao M Wang X Li Z. The effect of pension on labor participation of the young old in China. Popul Res. (2022) 4:69–83.

[62] Nie J Wu Y. Happiness from work or leisure? An empirical study of the influence of “no leisure/retirement” on rural Elderly’s sense of happiness. Jiang Han Acad. (2021) 5:60–71. doi: 10.16388/j.cnki.cn42-1843/c.2021.05.006

[63] Zhao Y Zhao Y Yuan X Zhou R. How knowledge contributor characteristics andreputation affect user payment decision in paid q&a? An empirical analysis firom theperspective of tust theoryJ. Electron Commer Res Appl. (2018) 31:1–11. doi: 10.1016/j.elerap.2018.07.001

[64] Mellor D Stokes M Firth L Hayashi Y Cummins R. Need for belonging, relationship satisfaction, loneliness, and life satisfaction. Pers Individ Differ. (2008) 45:213–8. doi: 10.1016/j.paid.2008.03.020, PMID: 38248125

[65] Baron RM Kenny DA. The moderator-mediator variable distinction in social psychological research: conceptual, strategic, and statistical considerations. J Pers Soc Psychol. (1986) 51:1173–82. doi: 10.1037/0022-3514.51.6.1173, PMID: 3806354

[66] Judd CM Kenny DA. Process analysis: estimating mediation in treatment evaluations. Eval Rev. (1981) 5:602–19. doi: 10.1177/0193841X8100500502, PMID: 38502955

[67] Wen Z Zhang L Hou J Liu H. Testing and application of the mediating effects. Acta Psychol Sin. (2004) 15:227–32. doi: 10.1007/BF02911031

[68] Wen Z Ye B. Analyses of mediating effects: the development of methods and models. Adv Psychol Sci. (2014) 22:731–45. doi: 10.3724/SP.J.1042.2014.00731

[69] Dahlen M Thorbjornsen H. Individuals' assessments of their own wellbeing, subjective welfare, and good life: four exploratory studies. Int J Environ Res Public Health. (2022) 19:19. doi: 10.3390/ijerph191911919, PMID: 36231218 PMC9565627

[70] Kapteyn A Lee J Zamarro G (2013). Does Retirement Induced through Social Security Pension Eligibility Influence Subjective Well-being? A Cross-Country Comparison. Working Papers wp301, University of Michigan, Michigan Retirement Research Center.

[71] Galiani S Gertler P Bando R. Non-contributory pensions. NBER Work Paper. (2014) 38:47–58. doi: 10.1016/j.labeco.2015.11.003

[72] Salinas-Rodríguez A Torres-Pereda MDP Manrique-Espinoza B Moreno-Tamayo K Téllez-Rojo Solís MM. Impact of the non-contributory social pension program 70 y más on older adults’ mental well-being. PLoS One. (2014) 9:1–10. doi: 10.1371/journal.pone.0113085, PMID: 25409468 PMC4237374

